# The Effect of Algae or Insect Supplementation as Alternative Protein Sources on the Volatile Profile of Chicken Meat

**DOI:** 10.3390/foods9091235

**Published:** 2020-09-04

**Authors:** Vasiliki Gkarane, Marco Ciulu, Brianne A. Altmann, Armin O. Schmitt, Daniel Mörlein

**Affiliations:** 1 Department of Animal Sciences, University of Göttingen, Kellnerweg 6, 37077 Göttingen, Germany; marco.ciulu@uni-goettingen.de (M.C.); brianne.altmann@agr.uni-goettingen.de (B.A.A.); daniel.moerlein@uni-goettingen.de (D.M.); 2 Breeding Informatics Group, Department of Animal Sciences, Georg-August University, Margarethe von Wrangell-Weg 7, 37075 Göttingen, Germany; armin.schmitt@uni-goettingen.de; 3 Center for Integrated Breeding Research (CiBreed), University of Göttingen, Albrecht-Thaer-Weg 3, 37075 Göttingen, Germany

**Keywords:** volatile compounds, sustainable feeds, spirulina, *Arthrospira platensis*, insect, *Hermetia illucens*

## Abstract

The aim of this study was to investigate the differences in the volatile profile of meat from chickens fed with alternative protein diets (such as algae or insect) through two different trials. In Trial 1, broiler chicken at one day of age were randomly allocated to three experimental groups: a basal control diet (C) and two groups in which the soybean meal was replaced at 75% (in the starter phase) and 50% (in the grower phase) with partially defatted *Hermetia illucens* (HI) larvae or *Arthrospira platensis* (SP). In Trial 2, broiler chickens were housed and reared similar to Trial 1, with the exception that the experimental diets replaced soybean meal with either 100% partially defatted HI or 100% SP. In both trials, chickens were slaughtered at day 35. Per group, 10 chickens were submitted to volatile analysis by using solid-phase microextraction (HS-SPME) and gas chromatography–mass spectrometry (GC-MS) analysis. Results in both trials showed that levels of several lipid-derived compounds were found to be lower in chickens fed an HI diet, which could be linked to a possibly lower level of polyunsaturated fatty acid content in HI-fed chicken. In addition, the dietary treatments could be discriminated based on the volatile profile, i.e., the substitution of soy with HI or SP distinctively affected the levels of flavor compounds.

## 1. Introduction

Intake of high-quality protein, in the range of 1.2–1.6 g per kg body weight per day, has been associated with health benefits, with poultry identified as one of the main sources [1]. Chicken meat consumption is popular for a number of reasons, not least of which are its nutritional value (i.e., the lower content of saturated fat and the higher proportion of polyunsaturated fatty acids (PUFA) compared to other types of meat), its affordable price, and its lack of religious and cultural constraints associated with its consumption [2].

Chicken meat composition and quality are highly associated with animal diet [3], which once supplemented with functional ingredients (e.g., antioxidants, n-3 PUFA) may enhance the nutritional value of meat [4]. To date, corn, soybean, and fishmeal are the main conventional components of poultry diets [5]. However, despite the high protein content and the well balanced amino acid profile that these ingredients provide [6,7], they are often linked with a negative environmental footprint (i.e., land degradation, water deprivation, greenhouse gas emissions, or marine overexploitation) [8]. In light of the forthcoming increase of the global population (expected to reach nine billion by 2050) [9], the rising consumer demand for high quality protein, along with the on-going environmental deterioration and climate change, there is an urgent need to switch to more sustainable feeds in animal production systems [10,11]. 

The use of microalga and insects have gained increasing attention in the last 30 years, holding the potential to act as alternative economical and more sustainable protein source for the livestock sector [12,13]. One of the most widely studied insect species is the black soldier fly (*Hermetia illucens*; HI), the larvae of which contains high levels of protein (approximately 45% dry matter (DM)) [14,15] and has an optimal amino acid composition [16]. In addition, studies in poultry meat show that growth performance, carcass traits, feeding efficiency [17,18], and several meat quality traits (pH, meat color, drip loss, and proximate or sensory analysis parameters) [18,19,20] are not negatively affected when soybean meal is partially or totally replaced by HI larvae.

*Arthrospira platensis* (commonly known as spirulina (SP)) is an edible microalga species with high nutritional value characterized by a 55–70% protein content [21]. Moreover, it has γ-linolenic acid, n-3 long chain, essential amino acids, vitamins, minerals, antioxidants, and carotenoids [22]. Khan et al. [23] summarizes the information available on the rich biological activity of spirulina including its antimicrobial, antioxidant, antiviral, anti-inflammatory, and immune-modulating role. Inclusion of microalgae in the poultry diet can affect the performance, oxidative stability, and meat quality parameters of broiler chickens [24,25,26].

Animal feeding strategies can modify the fatty acid profile of meat, as reflected in its volatile profile [27]. These interventions can influence the nutritional value, the eating quality, and consumer acceptance of meat [28]. Volatile compounds, formed mainly through after meat is cooked [29], have been widely used in meat provenance investigation studies as a way to trace, identify, or discriminate animal feeding systems [27,30]. Interestingly, differences in the volatile profile of chicken meat deriving from conventional vs. the aforementioned alternative protein sources have not yet been reported. Thus, the aim of this study was to investigate the volatile profile of chicken breast meat produced with a traditional soybean meal based diet (control, C diet) or diets with a partial substitution (75–50% in Trial 1) and complete substitution (100% in Trial 2) of soybean meal with either defatted HI or SP.

## 2. Materials and Methods 

### 2.1. Birds and Diet

The studies were carried out at the Department of Animal Sciences, University of Göttingen, and approved by the Ethics Committee of the Lower Saxony Office for Consumer Protection and Food Safety (LAVES; #33.9-42502-04-15/2027), Germany. The trials were part of a larger animal nutrition study, which investigated the effects of both alternative protein feeds on performance and apparent digestibility [31]. The trials were analyzed separately, as they were not exact replicates (i.e., substitution levels, spirulina source, sampling time, and storage temperature differed among the two trials). Ross-308 male broiler chickens were slaughtered at 35 days of age at the University of Göttingen poultry slaughterhouse, which is regulated by article 4 of the European Union’s (EG) NR. 853/2004 37. Immediately following slaughter, the carcasses were weighed and butchered, where the breasts (m. pectoralis major) were skinned and cooled to 4 °C (5 h), and then later frozen until further analysis. Average carcass weighed 1.77 ± 0.30 kg and 1.75 ± 0.24 kg for Trial 1 and Trial 2, respectively.

### 2.2. Trial 1 (75% to 50% Replacement)

The experiment was divided into a starter feeding phase (1–21 days) and a grower feeding phase (22–34 days). One-day-old chicks were randomly allocated to floor pens with 5.8 birds m^−2^ (i.e., 7 birds per pen). Average body weights per pen were similar at the start of the study. Feed and water were available ad libitum. The control diet (C) was based on wheat, corn, and soybean meal as the main ingredients. The experimental diets replaced soybean meal with either 75% partially defatted HI or SP in the starter phase and either 50% HI or SP in the grower phase. HI meal was sourced from a commercial producer (Hermetia Baruth GmbH, Baruth/Mark, Germany). The larvae were fattened on a rye and wheat bran substrate, dried at 65–70 °C, and partially-defatted with a screw press, then ground into a meal until an ultimate crude protein content 60.8% of DM and crude lipid content 14.1% of DM. Spirulina were sourced commercially from Myanmar, harvested, and sun-dried prior to packaging (crude protein content 58.8% of DM and crude lipid content 4.3% of DM). Amino acids were supplemented according to breeder guidelines. A total of 30 chicken breast samples were analyzed (10 C, 10 HI, and 10 SP). Samples originated from the chicken breast (m. pectoralis major) and were vacuum-packed in polyamide/polyethylene (PA/PE) bags and stored at −72 °C for 31 months until analysis. Full details of the animal and production characteristics and composition of feeds were described by Neumann et al. [31] (experiment 2).

### 2.3. Trial 2 (100% Replacement)

This experiment was also divided into a starter (1–21 days) and a grower feeding phase (22–34 days), and chicks were housed and reared as in Trial 1. One major modification is that the experimental diets replaced soybean meal with either 100% partially defatted HI or 100% SP in both feeding phases. HI meal was produced by a commercial producer (Hermetia Baruth GmbH, Baruth/Mark, Germany) with an ultimate crude protein content 60.8% of DM and crude lipid content 14.1% of DM. Spirulina were sourced commercially from China; the product was harvested, rinsed, and spray-dried prior to packaging (crude protein content 68.9% of DM and crude lipid content 6.3% of DM). A total of 30 chicken breast samples (10 C, 10 HI and 10 SP) were analyzed. Samples (m. pectoralis major) were vacuum-packed in PA/PE bags and stored at −20 °C for 26 months until analysis. Full details of the animal and production characteristics and composition of feeds were described by Neumann et al. [31] (experiment 3).

### 2.4. Sample Preparation and Volatile Compound Analysis

Volatile compounds were analyzed using headspace solid phase microextraction (HS-SPME). Before use, chicken breasts were thawed at room temperature by immersion of the frozen vacuum-packed samples in water, for 10 min. White tendons were removed and samples were minced by means of a mini chopper (DS Produkte GmbH, Gallin, Germany). Next, 4.5 ± 0.05 g of meat were weighed and transferred into 20 mL glass headspace vials together with 5 µL of a methanolic solution of 5 ppm 1,2-dichlorobenzene (Sigma-Aldrich, Munich, Germany), as internal standard. Vials were sealed with polytetrafluoroethylene (PTFE)-faced silicone septum (Macherey-Nagel, Düren, Germany). Meat samples were heated at 70 °C for 10 min in a TriPlus RSH^TM^ autosampler (Thermo Fisher Scientific, Waltham, MA, USA) for equilibration, before exposing the 30/50 μm Divinylbenzene/Carboxen/Polydimethylsiloxane (DVB/CAR/PDMS) fiber (Supelco, Bellefonte, PA, USA) into the headspace, where it was held for 20 min under constant stirring. Before its first use, the solid phase microextraction (SPME) fiber was thermally pre-conditioned at 270 °C for 1 h in accordance with the manufacturer recommendation. 

### 2.5. Gas Chromatography-Mass Spectrometry (GC-MS) Analyses

The GC–MS analyses of the samples were performed using a TRACE^TM^ 1310 gas-chromatograph coupled with an ISQ-LT single quadrupole mass spectrometer (Thermo Fisher Scientific, Waltham, MA, USA). The SPME fiber was thermally desorbed in a programmed temperature vaporizing injector at 250 °C in a splitless mode for 2 min while a split ratio of 1:20 was adopted for the remaining time of the chromatographic run. Inlet temperature was set at 250 °C while a desorption time of 7 min was adopted. Separation of compounds was achieved by a TG-5SILMS column (30 m × 0.25 mm × 0.25 µm) provided by Thermo Scientific. Helium was used as carrier gas operating at 1 mL min^−1^. Oven temperature was kept at 40 °C during the first 5 min, increased to 250 °C at 4 °C min^−1^, and was maintained at 250 °C during the last 2 min. After desorption, a fiber bake out was carried out in a bake out unit for 20 min at 260 °C to avoid carry-over phenomena among subsequent samples. The total chromatographic run time was 59.5 min. The MS detector operated in ion scan mode (45–230 amu), ion source, and transfer line temperature were maintained at 250 °C and 270 °C, respectively. The electron energy was 70 eV. Identification of the compounds was performed comparing (a) experimental mass spectra to those contained in the National Institute of Standards and Technology database (NIST/EPA/NIH Mass Spectral Library, Version 2.2, 2014) and (b) linear retention indexes (LRIs) based on a homologous series of n-alkanes (C7-C30, Sigma-Aldrich, Munich, Germany) with those reported in other studies. Data were expressed in ng per g meat using the formula provided by Wang et al. [32].

### 2.6. Data Analysis

GC–MS data were aligned per trial based on retention time in the R Core Team [33] using the package ‘CGalignR’ [34]. For a better match of slightly shifted peaks, a full join function provided by the package ‘fuzzyjoin’ [35] was applied, to allow for peak alignment until a maximum difference of 0.03 was achieved in both directions.

Volatile data did not meet the assumption of normal distribution. For this reason, the non-parametric Kruskal–Wallis test was applied in order to test the effect of dietary treatment on the volatile profile of chicken meat in each trial. In the case of a significant result of the Kruskal–Wallis test, the Wilcoxon rank-sum-test was applied to compare the medians of two groups. Differences were considered to be significant when *p* < 0.05. The results are presented as medians. Median absolute deviation was reported as measure of dispersion, defined as the median [absolute(x_i_ − median(x))], where x_i_ is an individual observation and median(x) is the median of values x. Linear discriminant analysis (LDA) was applied to investigate the potential separation of the dietary treatments within each trial [36] and to detect variables (compounds) that were more discriminant among feeding treatments. In order to detect the discriminatory efficiency of LDA, a confusion matrix followed by Cohen’s Kappa coefficient value was computed [37]. An individual animal was considered as the experimental unit.

## 3. Results

In total, 61 compounds in Trial 1 and 65 compounds in Trial 2, appearing in at least 60% of the samples (i.e., in 18 or more out of the 30 samples in each trial) were tentatively identified (Supplementary Table S1). The compounds that showed significant differences in the medians in Kruskal–Wallis test and the compounds constituting the minimal discriminatory set are marked in Table 1 (for Trial 1) and Table 2 (for Trial 2). Four compounds in each trial were not identified.

### 3.1. Volatile Compounds in Chicken Meat of Trial 1

In total, 210 volatile compounds were detected in chicken breast meat produced with the three different diets in Trial 1. The results of the Kruskal–Wallis test, presented in Table 1, indicate that 16 compounds showed significant difference among the treatments, 6 of which were detected in trace amounts (i.e., <0.30 ng/g). The main groups of compounds affected by diet were alcohols (4), aldehydes (6), ketones (1), hydrocarbons (2), lactones (1), while 2 compounds were not identified (Retention time (RT): 17.96 min and RT = 28.62 min). Two alcohols, 1-pentanol and 1-heptanol, were detected in lower levels in chicken breast meat produced with the HI diet compared to meat from the other two diets (*p* < 0.05), which did not differ from each other (*p* > 0.05). Levels of 1-hexanol, 1-octen-3-ol, hexanal and 2-heptenal, were lower under HI diet than under SP diet, neither of which differed from the C diet. In contrast, levels of 2-nonanone and pentadecane were higher in the HI diet compared to the other treatments, although levels were similar (*p* > 0.05) to the C diet and the SP diet, respectively.

**Figure 1:**
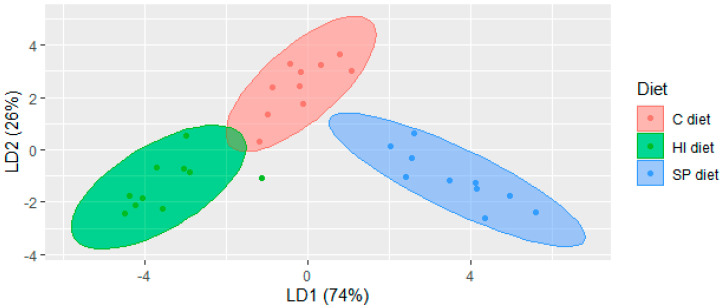
Linear Discriminant Analysis plot of the volatile compounds of chicken meat under three dietary treatments in Trial 1. C diet: Soybean meal- based diet; SP diet: Soybean meal-based diet partially supplemented with *Arthrospira platensis*; HI diet: Soybean meal-based diet partially supplemented with *Hermetia illucens* larvae.

Multivariate analysis was applied to investigate differences among the volatile profiles. LDA revealed that groups were clearly separated (Figure 1) with 18 compounds according to the confusion matrix and the kappa coefficient deriving from it. Four of these compounds were not identified (RTs: 17.96 min; 23.86 min; 28.62 min and 37.40 min; Supplementary Table S1) and were detected in traces. The first component (explaining 74% of the variation) separated mainly the HI group (located on the left lower quadrant) from the SP group (right lower quadrant). The compounds that contributed mostly in the separation (i.e., factor loadings higher than 1.0) were 1-pentanol, hexanal, 1-hexanol, 2-heptenal, 1-heptanol, 1-octen-3-ol, 2-nonanone, 2,4-decadienal, γ-nonalactone, pentadecane, heptadecane. The second component (explaining 26%) of the variation separated the C group (located on the upper side of the plot) from HI and SP group (bottom side of the plot). The compounds that contributed mostly to the separation (i.e., factor loadings higher than 1.0) were hexanal, 1-hexanol, 2-heptenal, 1-heptanol, 1-octen-3-ol, 2,4-decadienal and tridecanal (Supplementary Table S2).

### 3.2. Volatile Compounds in Chicken Meat of Trial 2

One hundred sixty-six volatile compounds were detected in the chicken breast meat of Trial 2. The results of the Kruskal–Wallis test (presented in Table 2) indicated that 33 compounds showed significant differences among treatments. The main groups of compounds that showed differences were alcohols (6), aldehydes (10), hydrocarbons (9), thiols (2), nitriles (1), and acids (1) while 4 compounds were not identified (unknown). In accordance with Trial 1, there were 7 compounds detected in trace amounts (i.e., <0.30 ng/g). Levels of 1-pentanol, 1-hexanol, 1-octen-3-ol, hexanal, heptanal, 2-heptenal, octanal, nonanal, decanal, 2,2,4,6-pentamethyl-heptane, decane, and one unknown (RT: 13.40 min) were lower (*p* < 0.05) in chickens from the HI-dietary treatment compared to SP-fed and C-fed chicken. Levels of 4-ethyl-cyclohexanol, 1-octanol, 2-nonen-1-ol, pentanal, toluene, 2,2,6-trimethyl-octane, undecane, 4-cyano-cyclohexene, and one unknown (RT: 18.15) were lower (*p* < 0.05) in chickens fed the HI-diet than the ones from C and/or the SP diet, which, in most cases, did not differ (*p* > 0.05). On the contrary, levels of 2-octenal, 2-undecenal, 4-methyl-nonane, 2,6,7-trimethyl-decane, 2-methyl-decane, 2,8-dimethyl-4-methylene-nonane, 2-ethyl-1-hexanethiol, 2-methyl-2-heptanethiol, 2-methyl-decane, and two unknown (RT:15.26 min and RT:16.51 min) were higher (*p* < 0.05) in HI-fed chickens in comparison to C-fed and/or the SP-fed chickens.

**Figure 2:**
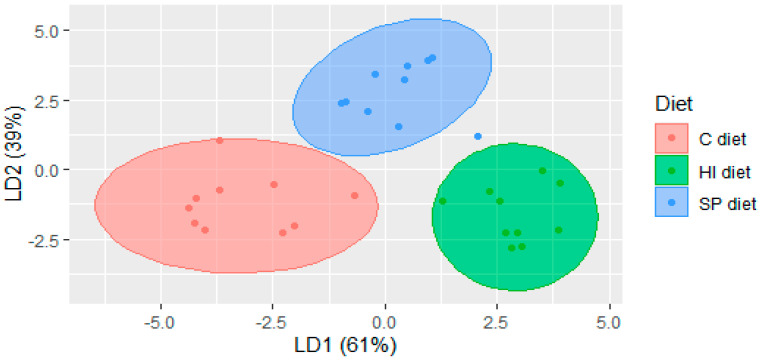
Linear discriminant analysis plot of the volatile compounds of chicken meat under three dietary treatments in Trial 2. C diet: Soybean meal-based diet; SP diet: Soybean meal-based diet supplemented with *Arthrospira platensis*; HI diet: Soybean meal-based diet supplemented with *Hermetia illucens* larvae.

Linear discriminant analysis of the volatile data separated the dietary groups of Trial 2 with 14 compounds according to the kappa-coefficient (Figure 2). The first component (explaining 61.1% of the variation) separated the HI group (left side of the plot) from the C group (right side of the plot). The compounds that contributed mostly in the separation (i.e., factor loadings higher than 1.0) were hexanal, 1-hexanol, heptanal, 2-heptenal, 4-methyl-nonane, 2,2,6-trimethyl-octane, 1-octen-3-ol, 2,6,7-trimethyl-decane, and 2-decenal. The second component explained 39% of the variation separated the SP group from the HI and C groups (Figure 2). The compounds that contributed to the separation were hexanal, 1-hexanol, heptanal, 2-heptenal, 4-methyl nonane, 2,2,6-trimethyl octane, 1-octen-3-ol, 2-ethyl-2-hexenol, 2,6,7-trimethyl-decane, 2-methyl-decane, and nonanal (Supplementary Table S3).

## 4. Discussion

Several studies have investigated the effect of dietary treatment on chicken meat quality [20,38,39,40]. Results indicated that the volatile profile was affected by dietary treatments, with the majority of the compounds having been reported in literature on chicken meat aroma (Supplementary Table S1). Chicken meat is prone to lipid oxidation due to its high content of PUFAs; thus, volatile compounds deriving from lipid oxidation (i.e., aldehydes, alcohols, and ketones) [41] were expected. 

Although a substantial number of compounds differed significantly among treatments of Trial 1, only half of them were detected in considerable quantities (i.e., higher than 0.30 ng/g). Results of Trial 2 indicated that the complete substitution of soybean meal with microalga or insects in the starter and grower phase strongly influenced the formation of lipid-derived compounds. Although acknowledging the fact that the two trials were independent and non-directly comparable, there was still a similar oxidation pattern observed among dietary treatments, i.e., the levels of compounds in meat produced with the HI-diet were usually lower than in the other dietary treatments, with most of the compounds deriving from linoleic acid (1-pentanol, 1-hexanol, 1-octen-3-ol, 2-nonen-1-ol, hexanal, heptanal, 2-heptenal) [42] or oleic acid (1-heptanol, 1-octanol, octanal, nonanal, decanal) [43].

The explanation of the results could be associated with the fatty acid (FA) profile of the diets since, in monogastric animals like chickens, the FA profile of the diet reflects the intramuscular FA composition (mainly the triglycerides, as the phospholipid composition is less affected [44]). The higher levels of the compounds in SP-fed and C-fed chicken compared to HI-fed chicken could be attributed to the possibly high level of PUFA in these diets [45,46,47,48], which could have promoted lipid oxidation. Cortinas Hernández et al. [38] attributed the linear increase of lipid oxidation in cooked chicken meat to the increasing PUFA content in raw meat. In this regard, Bonos, et al. [49] reported that dietary supplementation with 5 or 10 g spirulina per kg feed influenced the fatty acid composition of thigh muscle (by enhancing the PUFA content), but not of the breast muscle which had similar PUFA, saturated fatty acid (SFA), and mono unsaturated fatty acid (MUFA) content with the control diet. In contrast, El-Bahr et al. [24] reported that inclusion of 1 g spirulina per kg feed in a corn-soybean basal diet increased the major long chain n-3 PUFA (eicosapentaenoic, docosahexaenoic, and arachidonic acid) and total PUFA content in breast muscle, while the SFA, MUFA, and PUFA/SFA ratio remained unchanged.

In the case of an insect-based diet, the FA composition of chicken meat depends on the fatty acid profile of the insect lipids, which in turn may be affected by FA composition of the rearing substrate [14] or the stage in the life cycle of the insect [50]. HI larvae has been associated with high SFA content (ranging usually between 40–80%) and low PUFA content, i.e., around 10–20% of total FA [51] or even less, in larvae [52,53]. Thus, the lower extent of oxidation observed in muscle from HI-fed chicken could be associated with the reduced content of the easily oxidized PUFA and the elevated content of the (slowly oxidized) SFA in HI treatment. In support of this, Schiavone et al. [18] reported that 50% or 100% substitution of soybean oil with HI larvae increased the ratio of SFA of broiler chicken breast proportionally to the level of substitution and to the detriment of the PUFA fraction. The diminishing effect of HI larvae (meal or fat) on PUFA content was confirmed in Schiavone et al. [53] and Cullere et al. [54]. In addition, Schiavone et al. [53] noted that the defatting process not only enhances the protein content but may also reduce the risk of lipid oxidation in meat. Finally, volatile analysis showed that hexanal, a compound considered as an oxidation marker [55], was detected in lower levels under the HI diet than under the other two diets in both trials, which could imply less oxidation. Although the fatty acid profile of breast meat in our studies was not analyzed, a recent study by Altmann et al. [20] using the same animals as in Trial 1 reported a higher content of SFA in thigh muscle from chickens fed with the HI diet. It should be noted that lipid content between breast and thigh muscle in poultry meat differs [56] as well as the fatty acid composition [57]. As a consequence, the results of Altmann et al. [20] can only be seen as indicative. 

On the other hand, the higher levels of the two thiols in HI-fed chickens compared with the other two diets in Trial 2 could reflect differences in the free amino acid content among the three treatments, especially considering that thiol formation is linked with Strecker degradation of amino acids (e.g., cysteine) when reacting with secondary lipid oxidation compounds [58]. Differences in thiamine content may also play a role, as thiamine degradation is another pathway for thiol formation [59].

It appears that several factors could be involved in the extent of lipid oxidation when feeds of high nutritional value, like microalga or insects, are included in poultry diets. These factors may be related to the type of microalga [24] or insect species [60], the level of supplementation, the stage of inclusion in the basal diets (starter, grower, finisher phase), the type and level of antioxidants (e.g., β-carotene, tocopherol, carotenoids) that microalga contain and may affect the oxidative stability [61], the type of substrate on which larvae were reared [14], the use of defatted biomasses (algae or insect) [62,63], the defatting method [64], or the interaction with other factors, like amino acid supplementation [24,65].

Overall, the results of the two trials indicate that alternative protein sources may affect the aromatic profile of chicken meat and this could impact sensory perception. For example, hexanal (detected in the highest abundance in both trials) is associated with a “green/grassy” odor [66] and has been identified as one of the most important odor active compounds in chicken breast on the basis of its odor activity value [67]. 2-Heptenal, heptanal, octanal, nonanal, and decanal (described as “oily/fatty”, “fatty/roasty/citrus”, “fatty/sweet”, “roasted/meaty/fatty”, and “sweet/fruity”, respectively [66,68]) due to their low odor threshold values [69] and the relatively high level at which they were detected in C and SP diets, mainly in Trial 2, could potentially affect aroma or flavor perception in chickens fed these diets. Although the significant alcohols have been previously reported in chicken aroma studies, only 1-octen-3-ol, with the characteristic “mushroom” odor [68], has a low odor threshold (1 ng/g) compared to the other alcohols [69,70] and was detected in significant quantities in both trials. Hydrocarbons are derived from lipid oxidation and are compounds that showed significant variations among treatments. However, these are generally not considered to impact the flavor of lipid-based foods [71]. Thiols are significant volatile compounds in meat aroma [72]. The higher levels of the two thiols in HI-fed chicken compared with the other two diets in Trial 2 could signify differences in the aromatic quality of the three diets. As meat flavor is a combination of volatile aroma and non-volatile taste compounds, further analytical research (e.g., defining free amino acid content; performing gas chromatography-olfactometry analysis) in combination with sensory evaluation would be required to identify compounds that could influence eating quality and possibly consumer preference.

LDA indicated the potential of the volatile analysis/profile to discriminate the three dietary treatments in both trials. Compounds that had higher impact on the group separation were mainly those with higher concentration, rather than compounds that were only detected in traces. The content of linoleic acid—the precursor of most of these compounds—seemed to play a significant role in the characterization and identification of these treatments.

## 5. Conclusions

This is the first study to report differences in the volatile profile of chicken meat after dietary replacement of soybean meal with alternative protein sources (i.e., microalga or partially defatted larvae meal). The differences detected were mainly in the lipid oxidation-deriving compounds that could play a significant role in the development of the typical aroma of chicken meat. Multivariate analysis confirmed that the dietary treatments led to a discriminatory volatile profile in both trials. Considering that both microalga and insects could stand as sustainable options in animal feeding in the years to come, future research should focus on identifying the type of biomasses and the proper inclusion level in order to improve chicken meat quality.

## Figures and Tables

**Table 1:** The composition of the volatile profile of chicken breast meat fed with three different diets in Trial 1 ((Control (C) vs. Spirulina (SP) vs. *Hermetia illucens* (HI)) (results expressed as ng/g).

	C ^1^	SP ^1^	HI ^1^	Statistical Analysis	*p*-Level
Alcohols					
2-methoxy-ethanol	9.36 (13.17)	9.50 (5.57)	8.45 (3.60)		
1-Penten-3-ol	4.48 (9.87)	6.15 (5.33)	7.86 (4.12)		
1-Pentanol	2.13 ^a^ (3.17)	2.54 ^a^ (1.95)	0. 11 ^b^ (0.11)	KW; LDA	0.026
1-Hexanol	1.26 ^ab^ (1.57)	1.40 ^a^ (0.59)	0.71 ^b^ (0.23)	KW; LDA	0.030
1-Heptanol	0.87 ^a^ (0.92)	0.73 ^a^ (0.31)	0.32 ^b^ (0.08)	KW; LDA	0.027
1-Octen-3-ol	21.3 ^ab^ (21.6)	31.4 ^a^ (10.1)	13.2 ^b^ (1.77)	KW; LDA	0.034
2-Ethyl-2-hexenol	4.02 (4.13)	2.17 (0.86)	1.57 (0.85)		
4-Ethyl-cyclohexanol	0.00 (0.53)	0.00 (0.00)	0.00 (0.00)		
2-Ethyl-1-hexanol	4.70 (6.76)	5.26 (3.16)	4.41 (3.15)		
2-Ethyl-1-decanol	1.11 (1.11)	0.77 (0.40)	0.51 (0.21)		
2-Octen-1-ol	0.75 (0.77)	0.88 (0.47)	0.34 (0.24)		
1-Octanol	4.02 (4.25)	3.19 (0.88)	2.91 (0.77)		
Benzenemethanol, α, α-dimethyl	0.61 (0.78)	0.46 (0.34)	0.56 (0.51)		
(*Z*)-2-Nonen-1-ol	1.14 (1.01)	0.79 (0.38)	0.54 (0.20)		
1-Nonanol	0.21 (0.25)	0.25 (0.07)	0.26 (0.16)		
Aldehydes					
Pentanal	4.16 (7.53)	7.08 (4.95)	3.52 (3.52)		
Hexanal	148.1 ^ab^ (143.4)	164.3 ^a^ (42.8)	78.62 ^b^ (41.7)	KW; LDA	0.043
Heptanal	3.48 (4.33)	4.89 (1.46)	2.94 (1.13)		
Methional	nd	nd	nd		
2-Heptenal	0.52 ^ab^ (1.12)	0.75 ^a^ (0.45)	0.13 ^b^ (0.13)	KW; LDA	0.022
Benzaldehyde	0.49 (0.66)	0.00 (0.00)	1.0 (0.80)		
Octanal	7.91 (8.01)	7.74 (3.00)	5.98 (1.07)		
2-Octenal	0.84 (1.09)	1.01 (0.45)	0.70 (0.37)		
Nonanal	23.8 (18.2)	23.7 (10.1)	17.5 (5.73)		
(*E*)-2-Nonenal	0.30 (0.32)	0.38 (0.14)	0.24 (0.05)		
(*Z*)-4-Decenal	0.00 ^a^ (0.00)	0.00 ^a^ (0.00)	0.00 ^b^ (0.00)	KW; LDA	0.026
Decanal	0.96 (1.37)	1.37 (0.74)	0.89 (0.49)		
2-Decenal	0.20 (0.22)	0.29 (0.13)	0.15 (0.05)		
(*E,E*)-2,4-Decadienal	0.00 ^b^ (0.18)	0.17 ^a^ (0.11)	0.06 ^ab^ (0.06)	KW; LDA	0.028
(*E*)-2-Undecenal	0.10(0.12)	0.12 (0.05)	0.06 (0.02)		
Dodecanal	0.24 (0.24)	0.30 (0.12)	0.20 (0.06)		
Tridecanal	0.04 ^ab^ (0.05)	0.07 ^a^ (0.04)	0.01 ^b^ (0.01)	KW; LDA	0.029
Tetradecanal	0.06 ^ab^ (0.06)	0.08 ^a^ (0.03)	0.03 ^b^ (0.03)	KW; LDA	0.031
Ketones					
2-Heptanone	0.79 (1.03)	0.81 (0.34)	0.83 (0.23)		
Butyrolactone	nd	nd	nd		
2-Methyl-6-heptanone	0.13 (0.55)	0.03 (0.03)	0.00 (0.00)		
2-Nonanone	0.08 ^ab^ (0.12)	0.00 ^b^ (0.00)	0.38 ^a^ (0.32)	KW; LDA	0.005
Hydrocarbons					
Toluene	nd	nd	nd		
1,2,4-Trimethyl-cyclopentane	nd	nd	nd		
Propyl-cyclohexane	0.00 (2.16)	0.00 (0.00)	0.00 (0.00)		
4-Methyl-nonane	nd	nd	nd		
2,2,6-Trimethyl-octane	nd	nd	nd		
2,2,4,6-Pentamethyl-heptane	40.4 (33.5)	39.7 (16.0)	27.2 (8.66)		
Decane	0.41 (0.42)	0.29 (0.24)	0.18 (0.14)		
2,2,4,4-Tetramethyl-octane	3.16 (3.42)	2.40 (1.05)	2.23 (1.06)		
2,6,7-Trimethyl-decane	0.67 (0.63)	0.51 (0.35)	0.34 (0.34)		
2-Methyl-decane	0.00 (0.09)	0.00 (0.00)	0.00 (0.00)		
5-Undecene	nd	nd	nd		
Undecane	1.10 (1.18)	1.09 (0.21)	1.02 (0.42)		
2,8-Dimethyl-4-methylene-nonane	nd	nd	nd		
Pentyl-cyclohexane	nd	nd	nd		
3-Methylene-undecane	0.00 (0.07)	0.00 (0.00)	0.00 (0.00)		
Dodecane	1.16 (1.23)	1.22 (0.45)	0.95 (0.39)		
2,6,11-Trimethyl-dodecane	0.17 (0.19)	0.30 (0.10)	0.23 (0.09)		
Tridecane	0.55 (0.62)	0.79 (0.35)	0.58 (0.20)		
2,3,5,8-Tetramethyl-decane	0.19 (0.19)	0.23 (0.08)	0.20 (0.06)		
Tetradecane	0.52 (0.50)	0.61 (0.22)	0.48 (0.14)		
Pentadecane	0.36 ^b^ (0.66)	0.57 ^ab^ (0.13)	1.18 ^a^ (0.75)	KW; LDA	0.013
2,6,10-Trimethyl-tetradecane	0.07 (0.08)	0.10 (0.03)	0.08 (0.05)		
Hexadecane	0.21 (0.21)	0.28 (0.06)	0.20 (0.06)		
Heptadecane	0.03 ^b^ (0.03)	0.28 ^a^ (0.10)	0.00 ^b^ (0.00)	KW; LDA	0.001
Thiols					
2-Ethyl-1-hexanethiol	nd	nd	nd		
2-Methyl-2-heptanethiol	nd	nd	nd		
Esters					
Pentanoic acid,2,2,4-trimethyl-3-hydroxy-, isobutyl ester	0.06 (0.09)	0.08 (0.08)	0.15 (0.09)		
Carbamodithioic acid, diethyl-, methyl ester	0.24 (0.27)	0.35 (0.14)	0.14 (0.14)		
Dimethyl phtalate	4.42 (4.74)	5.73 (1.43)	4.70 (0.92)		
Pentanoic acid, 2,2,4-trimethyl-3-carboxyisopropy, isobutyl ester	0.00 (0.01)	0.00 (0.00)	0.00 (0.00)		
Lactone					
γ-nonalactone	0.12 ^b^ (0.12)	0.20 ^a^ (0.07)	0.09 ^b^ (0.03)	KW; LDA	0.046
Acid					
Dodecanoic acid	0.00 (0.01)	0.00 (0.02)	0.00 (0.00)		
Nitrile					
4-Cyano-cyclohexene	0.00 (0.23)	0.00 (0.00)	0.16 (0.16)		
Azide					
2-Azido-2,4,4,6,6-pentamethyl-heptane	0.15 (0.26)	0.05 (0.05)	0.15 (0.11)		
Unknown					
Unknown (RT:17.96 min)	0.00 ^b^ (0.00)	0.00 ^b^ (0.00)	0.00 ^a^ (0.00)	KW; LDA	0.040
Unknown (RT:23.76 min)	0.01 (0.03)	0.00 (0.00)	0.09 (0.07)	LDA	
Unknown (RT:28.62 min)	0.00 ^a^ (0.00)	0.00 ^b^ (0.00)	0.00 ^a^ (0.00)	KW; LDA	0.012
Unknown (RT:37.40 min)	0.00 (0.02)	0.00 (0.00)	0.00 (0.00)	LDA	

^1^, Values are expressed as: Median (Median absolute deviation); ^a, b^, medians assigned different superscripts indicate significant differences (*p* < 0.05) between the dietary treatments; KW: Compounds that were found to be significantly different (*p* < 0.05) due to different dietary treatment, following the Kruskal–Wallis Test; LDA: Compounds that belong to the minimal set of 18 compounds that lead to a complete separation of the three dietary groups in linear discriminant analysis, i.e., to a confusion matrix with kappa = 1; nd: not detected.

**Table 2:** The composition of the volatile profile of chicken breast meat fed with three different diets in Trial 2 ((Control (C) vs. Spirulina (SP) vs. *Hermetia illucens* (HI)) (results expressed as ng/g).

	C ^1^	SP ^1^	HI ^1^	Statistical Analysis	*p*-Level
Alcohols					
2-Methoxy-ethanol	3.43 (2.11)	1.62 (1.58)	1.71 (1.10)		
1-Penten-3-ol	3.98 (2.84)	2.93 (1.30)	2.58 (2.49)		
1-Pentanol	8.88 ^a^ (5.76)	9.89 ^a^ (3.72)	2.02 ^b^ (1.08)	KW	0.016
1-Hexanol	4.20 ^a^ (2.59)	4.69 ^a^ (2.72)	1.53 ^b^ (0.38)	KW; LDA	0.003
1-Heptanol	1.44 (0.83)	2.49 (0.86)	1.52 (0.49)		
1-Octen-3-ol	30.8 ^a^ (13.7)	36.0 ^a^ (9.44)	11.6 ^b^ (0.93)	KW; LDA	0.001
2-Ethyl-2-hexenol	nd	nd	nd		
4-Ethyl-cyclohexanol	4.39 ^ab^ (4.39)	6.30 ^a^ (1.65)	2.53 ^b^ (0.60)	KW	0.009
2-Ethyl-1-hexanol	12.2 (3.02)	19.4 (4.95)	20.3 (2.75)		
2-Ethyl-1-decanol	3.38 (1.66)	3.90 (1.99)	4.78 (1.32)		
2-Octen-1-ol	0.32 (0.32)	1.34 (1.34)	1.53 (0.73)		
1-Octanol	4.05 ^ab^ (2.02)	4.83 ^a^ (1.15)	2.61 ^b^ (0.71)	KW	0.045
Benzenemethanol,α,α-dimethyl	0.21 (0.21)	0.81 (0.37)	0.68 (0.20)		
(*Z*)-2-Nonen-1-ol	4.80 ^ab^ (1.53)	6.52 ^a^ (1.95)	4.17 ^b^ (1.06)	KW	0.041
1-Nonanol	0.05 (0.05)	0.24 (0.24)	0.24 (0.16)		
Aldehydes					
Pentanal	8.04 ^a^ (3.71)	7.40 ^ab^ (2.21)	2.85 ^b^ (1.23)	KW	0.026
Hexanal	283.7 ^a^ (135.8)	230.5 ^a^ (123.7)	44.7 ^b^ (20.8)	KW; LDA	0.001
Heptanal	5.64 ^a^ (2.44)	9.90 ^a^ (3.12)	2.30 ^b^ (0.51)	KW; LDA	0.002
Methional	0.20 (0.20)	0.08 (0.08)	0.27 (0.15)		
2-Heptenal	0.71 ^a^ (0.42)	0.54 ^a^ (0.25)	0.08 ^b^ (0.07)	KW; LDA	0.001
Benzaldehyde	3.07 (1.31)	2.71 (0.57)	3.14 (0.84)		
Octanal	16.0 ^a^ (6.11)	15.8 ^a^ (6.35)	6.18 ^b^ (1.36)	KW; LDA	0.004
2-Octenal	1.23 ^b^ (0.68)	3.02 ^ab^ (1.22)	3.50 ^a^ (1.13)	KW	0.045
Nonanal	24.8 ^a^ (8.06)	31.6 ^a^ (8.50)	11.8 ^b^ (2.52)	KW; LDA	0.008
(*E*)-2-Nonenal	0.53 (0.26)	0.54 (0.13)	0.33 (0.05)		
(*Z*)-4-Decenal	0.00 (0.00)	0.28 (0.10)	0.23 (0.09)		
Decanal	0.87 ^a^ (0.25)	0.96 ^a^ (0.37)	0.09 ^b^ (0.09)	KW	0.008
(*E*)-2-Decenal	0.06 ^ab^ (0.06)	0.19 ^a^ (0.07)	0.00 ^b^ (0.00)	KW; LDA	0.003
(*E,E*)-2,4-Decadienal	0.06 (0.06)	0.09 (0.04)	0.03 (0.03)		
(*E*)-2-Undecenal	0.00 ^ab^ (0.00)	0.07 ^b^ (0.07)	0.00 ^a^ (0.00)	KW	0.022
Dodecanal	0.05 (0.05)	0.04 (0.04)	0.00 (0.00)		
Tridecanal	nd	nd	nd		
Tetradecanal	nd	nd	nd		
Ketones					
2-Heptanone	0.35 (0.31)	0.28 (0.18)	0.21 (0.08)		
Butyrolactone	0.42 (0.40)	0.97 (0.72)	0.24 (0.16)		
2-Methyl-6-heptanone	0.00 (0.00)	0.00 (0.00)	0.00 (0.00)		
2-Nonanone	0.00 (0.00)	0.00 (0.00)	0.00 (0.00)		
Hydrocarbons					
Toluene	0.17 ^a^ (0.17)	0.03 ^ab^ (0.03)	0.00 ^b^ (0.00)	KW	0.048
1,2,4-Trimethyl-cyclopentane	0.68 (0.68)	0.32 (0.23)	0.16 (0.16)		
Propyl-cyclohexane	0.35 (0.28)	0.34 (0.07)	0.27 (0.08)		
4-Methyl-nonane	0.00 ^b^ (0.00)	0.00 ^b^ (0.00)	0.22 ^a^ (0.16)	KW; LDA	0.002
2,2,6-trimethyl-octane	0.00 ^a^ (0.00)	0.00 ^ab^ (0.00)	0.48 ^b^ (0.48)	KW; LDA	0.004
2,2,4,6-Pentamethyl-heptane	53.5 ^a^ (35.0)	25.8 ^a^ (4.86)	19.3 ^b^ (6.90)	KW	0.011
Decane	4.24 ^a^ (2.96)	2.43 ^a^ (1.59)	1.50 ^b^ (0.44)	KW	0.034
2,2,4,4-Tetramethyl-octane	4.34 (1.86)	1.24 (1.24)	0.73 (0.73)		
2,6,7-Trimethyl-decane	1.43 ^b^ (1.43)	8.24 ^a^ (2.20)	10.8 ^a^ (3.84)	KW; LDA	0.000
2-Methyl-decane	0.00 ^b^ (0.00)	2.42 ^a^ (0.95)	3.18 ^a^ (1.27)	KW; LDA	0.000
5-Undecene	0.29 (0.29)	2.37 (1.33)	2.50 (1.09)		
Undecane	1.57 ^a^ (1.46)	0.12 ^b^ (0.08)	0.09 ^b^ (0.04)	KW	0.024
2,8-dimethyl-4-methylene-nonane	0.00 ^b^ (0.00)	0.07 ^ab^ (0.07)	0.20 ^a^ (0.19)	KW	0.029
Pentyl-cyclohexane	0.28 (0.24)	0.39 (0.12)	0.64 (0.21)		
3-Methylene-undecane	0.25 (0.23)	0.26 (0.14)	0.39 (0.14)		
Dodecane	0.93 (0.79)	0.15 (0.14)	0.09 (0.09)		
2,6,11-trimethyl-dodecane	0.05 (0.05)	0.03 (0.03)	0.00 (0.00)		
Tridecane	0.50 (0.29)	0.28 (0.07)	0.25 (0.11)		
2,3,5,8-tetramethyl-decane	0.02 (0.02)	0.00 (0.00)	0.01 (0.01)		
Tetradecane	0.30 (0.09)	0.32 (0.07)	0.22 (0.05)		
Pentadecane	0.21 (0.08)	0.24 (0.10)	0.24 (0.09)		
2,6,10-trimethyl-tetradecane	0.00 (0.00)	0.00 (0.00)	0.00 (0.00)		
Hexadecane	0.12 (0.07)	0.14 (0.08)	0.08 (0.03)		
Heptadecane	0.00 (0.00)	0.05 (0.05)	0.00 (0.00)		
Thiols					
2-Ethyl-1-hexanethiol	0.58 ^b^ (0.58)	5.86 ^a^ (1.33)	8.94 ^a^ (3.24)	KW	0.009
2-Methyl-2-heptanethiol	4.48 ^b^ (1.00)	7.29 ^ab^ (1.64)	9.68 ^a^ (2.53)	KW	0.009
Esters					
Pentanoic acid,2,2,4-trimethyl-3-hydroxy-, isobutyl ester	0.00 (0.00)	0.00 (0.00)	0.00 (0.00)		
Carbamodithioic acid, diethyl-, methyl ester	nd	nd	nd		
Dimethyl phtalate	1.85 (0.53)	1.93 (0.95)	1.35 (0.41)		
Pentanoic acid, 2,2,4-trimethyl-3-carboxyisopropyl, isobutyl ester	0.04 (0.04)	0.10 (0.06)	0.07 (0.04)		
Lactone					
γ-Nonalactone	nd	nd	nd		
Acid					
Dodecanoic acid	0.00 (0.00)	0.00 (0.00)	0.00 (0.00)	KW	0.040
Nitrile					
4-Cyano-cyclohexene	4.35 ^a^ (2.31)	0.98 ^ab^ (0.98)	0.31 ^b^ (0.31)	KW	0.041
Azide					
2-Azido-2,4,4,6,6-pentamethyl-heptane	nd	nd	nd		
Unknown					
Unknown (RT = 13.40 min)	0.35 ^a^ (0.22)	0.57 ^a^ (0.33)	0.00 ^b^ (0.00)	KW; LDA	0.006
Unknown (RT = 15.26 min)	1.78 ^b^ (1.06)	8.07 ^a^ (2.91)	10.2 ^a^ (4.58)	KW; LDA	0.000
Unknown (RT = 16.51 min)	0.30 ^a^ (0.30)	0.00 ^b^ (0.00)	0.00 ^ab^ (0.00)	KW	0.020
Unknown (RT = 18.15 min)	0.26 ^ab^ (0.26)	0.30 ^a^ (0.16)	0.00 ^b^ (0.00)	KW	0.045

^1^, Values presented as: Median (Median absolute deviation); ^a, b^, medians assigned different superscripts indicate significant differences (*p* < 0.05) between the dietary treatments; KW: Compounds that were found to be significantly different (*p* < 0.05) due to different dietary treatment, following the Kruskal–Wallis Test; LDA: Compounds that belong to the minimal set of 14 compounds that lead to a complete separation of the three dietary groups in linear discriminant analysis, i.e., to a confusion matrix with kappa = 1; nd: not detected.

## Supplementary Materials

The following are available online at https://www.mdpi.com/2304-8158/9/9/1235/s1, Table S1: Linear retention indices (LRIs) and method of identification of the main compounds detected in chicken meat (Trial 1 and Trial 2); Table S2: Factor loadings of the 18 most discriminant compounds of Trial 1 that lead to a confusion matrix with a Cohen’s kappa coefficient value of 1.0, i.e., clear separation of the three groups, Table S3: Factor loadings of the 14 most discriminant compounds of Trial 2 that lead to a confusion matrix with a Cohen’s kappa coefficient value of 1.0, i.e., clear separation of the three groups.
