# Supplementary material for: The Effect of Algae or Insect Supplementation as Alternative Protein Sources on the Volatile Profile of Chicken Meat
Source: Foods. 2020 Sep 4;9(9):1235. doi: 10.3390/foods9091235 (PMC7555012; doi:10.3390/foods9091235)
Supplement: Supplementary file 1 [file foods-09-01235-s001.zip › Supplementary Table S2.docx]

Supplementary Table S2. Factor loadings of the 18 most discriminant compounds of Trial 1 that lead to a confusion matrix with a Cohen's kappa coefficient value of 1.0, i.e. clear separation of all three groups.

| Compound | LD1 | LD2 |
| --- | --- | --- |
|  |  |  |
| 1-Pentanol | 1.28 | 0.38 |
| Hexanal | 5.40 | -5.18 |
| Hexanol | -2.85 | 2.62 |
| 2-Heptenal | -8.13 | -2.24 |
| 1-Heptanol | 1.42 | -5.77 |
| 1-Octen-3-ol | 4.83 | 6.97 |
| Unknown (RT: 17.96 min) | -0.11 | 0.16 |
| 2-Nonanone | -1.82 | 0.83 |
| 4-Decenal | -0.76 | -0.34 |
| Unknown (RT: 23.76 min) | 0.27 | 0.40 |
| 2,4-Decadienal | 2.10 | 3.94 |
| (γ-nonalactone) | -1.76 | 0.19 |
| Unknown (RT:28.62 min) | 0.81 | 0.66 |
| Pentadecane | -1.46 | -0.11 |
| Tridecanal | -0.13 | -1.12 |
| Tetradecanal | -0.05 | 0.19 |
| Unknown (RT:37.40 min) | 0.02 | -0.77 |
| Heptadecane | 2.21 | 0.58 |
