# Supplementary material for: The Effect of Algae or Insect Supplementation as Alternative Protein Sources on the Volatile Profile of Chicken Meat
Source: Foods. 2020 Sep 4;9(9):1235. doi: 10.3390/foods9091235 (PMC7555012; doi:10.3390/foods9091235)
Supplement: Supplementary file 1 [file foods-09-01235-s001.zip › Supplementary Table S3.docx]

Supplementary Table 3. Factor loadings of the 14 most discriminant compounds of Trial 2 that lead to a confusion matrix with a Cohen's kappa coefficient value of 1.0, i.e. clear separation of all three groups.

| Compound | LD1 | LD2 |
| --- | --- | --- |
| Hexanal | 2.81 | 2.06 |
| 1-Hexanol | -5.29 | -2.06 |
| Heptanal | 2.00 | 4.02 |
| 2-Heptenal | -1.70 | -4.26 |
| 4-Methyl-nonane | -1.19 | -2.17 |
| 2,2,6-Trimethyl-octane | 2.32 | 1.38 |
| Unknown (RT:13.40 min) | 3.31 | 0.27 |
| 1-Octen-3-ol | 3.79 | 4.58 |
| Octanal | -0.93 | -1.32 |
| Unknown (RT: 15.26 min) | -0.07 | -3.27 |
| 2,6,7-Trimethyl-decane | -3.12 | 2.51 |
| 2-Methyl-decane | 0.16 | 1.29 |
| Nonanal | -0.56 | -1.40 |
| 2-Decenal | -2.22 | -0.89 |
