# Supplementary material for: The Effect of Algae or Insect Supplementation as Alternative Protein Sources on the Volatile Profile of Chicken Meat
Source: Foods. 2020 Sep 4;9(9):1235. doi: 10.3390/foods9091235 (PMC7555012; doi:10.3390/foods9091235)
Supplement: Supplementary file 1 [file foods-09-01235-s001.zip › Supplementary Table S1.doc]

Supplementary Table 1. Linear retention indices (LRIs) and method of identification of the main compounds detected in chicken meat (Trial 1 and Trial 2).

|  | LRI^a^(exp) | LRI^b^(lit) | Method of identification^c^ | References to studies reporting the same compounds on chicken meat |
| --- | --- | --- | --- | --- |
| Alcohols |  |  |  |  |
| 2-Methoxy-ethanol |  | 624 ^[33, 60]^ | NIST |  |
| 1-Penten-3-ol |  | 684^[15]^ | LRI, NIST | 2, 34, 40, 44 |
| 1-Pentanol | 767 | 773 ^[4, 5, 36]^ | LRI, NIST | 11, 34, 37, 44, 66, |
| 1-Hexanol | 874 | 871 ^[5, 56, 64]^ | LRI, NIST | 34, 40, 59 |
| 1-Heptanol | 974 | 968 ^[15, 25, 53]^ | LRI, NIST | 34, 44, 66 |
| 1-Octen-3-ol | 982 | 980 ^[18, 57, 64]^ | LRI, NIST | 2, 3, 9, 11, 41 |
| 2-Ethyl-2-hexenol | 998 | 990 ^[29, 60]^ | LRI, NIST |  |
| 4-Ethyl-cyclohexanol | 1009 | 1003 ^[60]^ | LRI, NIST |  |
| 2-Ethyl-1-hexanol | 1032 | 1030 ^[18, 63, 64]^ | LRI, NIST | 9, 40, 45, 59 |
| 2-Ethyl-1-decanol | 1048 | 1052 ^[1]^ | LRI, NIST |  |
| 2-Octen-1-ol | 1069 | 1067 ^[28, 52, 53]^ | LRI, NIST | 40 |
| 1-Octanol | 1073 | 1072 ^[4, 12, 28]^ | LRI, NIST | 9, 11, 34, 40, 42, 59 |
| Benzenemethanol,α,α-dimethyl- | 1085 | 1085 ^[19, 21, 58]^ | LRI, NIST | 59 |
| 2-Nonen-1-ol (Z) | 1114 | 1114 ^[18, 52]^ | LRI, NIST |  |
| 1-Nonanol | 1171 | 1176 ^[28, 31, 39]^ | LRI, NIST |  |
|  |  |  |  |  |
| Aldehydes |  |  |  |  |
| Pentanal | 703 | 700 ^[37, 38, 62]^ | LRI, NIST | 42, 46, 48, 61, 66 |
| Hexanal | 802 | 801 ^[31, 41, 52]^ | LRI, NIST | 9, 14, 66 |
| Heptanal | 903 | 903 ^[18,51, 57]^ | LRI, NIST | 3, 14, 40 |
| Methional | 908 | 907 ^[15, 22, 28]^ | LRI, NIST | 13, 14, 22 |
| 2-Heptenal | 958 | 954 ^[36, 53, 57]^ | LRI, NIST | 3,13, 40, 49 |
| Benzaldehyde | 960 | 959 ^[38, 55, 56]^ | LRI, NIST | 26, 34, 44,66 |
| Octanal | 1003 | 1001 ^[4, 56, 38]^ | LRI, NIST | 11, 14, 66 |
| 2-Octenal | 1058 | 1061 ^[10, 57, 64]^ | LRI, NIST | 11, 37, 42, 66 |
| Nonanal | 1104 | 1105 ^[12, 20, 41]^ | LRI, NIST | 11, 14, 34, 37, 42, 43, 44 |
| 2-Nonenal (E) | 1159 | 1161 ^[10, 28, 39,]^ | LRI, NIST | 9, 34, 42, 66 |
| 4-Decenal (Z) | 1192 | 1195 ^[37, 60, 67]^ | LRI, NIST | 37 |
| Decanal | 1205 | 1206 ^[23, 57, 64]^ | LRI, NIST | 2, 3, 9, 13, 59 |
| 2-Decenal (E) | 1261 | 1264 ^[36, 26, 28]^ | LRI, NIST | 11, 13, 34, 42, 49 |
| 2,4-Decadienal (E,E) | 1317 | 1316 ^[31, 41, 64]^ | LRI, NIST | 3, 9, 13, 22, 40, 49 |
| 2-Undecenal (E) | 1364 | 1368 ^[28, 41]^ | LRI, NIST | 9, 11, 13, 34, 48, 41 |
| Dodecanal | 1409 | 1411 ^[15, 57, 63]^ | LRI, NIST | 42 |
| Tridecanal | 1510 | 1511 ^[15, 19, 36]^ | LRI, NIST | 35 |
| Tetradecanal | 1612 | 1610 ^[19, 36, 63]^ | LRI, NIST | 48, 49 |
|  |  |  |  |  |
| Ketones |  |  |  |  |
| 2-Heptanone | 892 | 888 ^[15, 38, 64]^ | LRI, NIST | 9, 42, 44, |
| Butyrolactone | 915 | 916 ^[4, 36, 47]^ | LRI, NIST | 9, 42 |
| 2-Methyl, 6-heptanone | 956 | 956 ^[17, 27]^ | LRI, NIST | 35 |
| 2-Nonanone | 1092 | 1091 ^[4, 15, 21]^ | LRI, NIST | 14, 42, 66, |
|  |  |  |  |  |
| Hydrocarbons |  |  |  |  |
| Toluene | 760 | 762^[12,15,18]^ | LRI, NIST | 42, 48 |
| 1,2,4-Trimethyl-cyclopentane | 788 | 779 ^[62]^ | LRI, NIST |  |
| Propyl-cyclohexane | 930 | 928 ^[24]^ | LRI, NIST |  |
| 4-Methyl-nonane | 967 | 963 ^[24, 25]^ | LRI, NIST |  |
| 2,2,6-trimethyl-octane, | 976 | 973 ^[5]^ | LRI, NIST | 37 |
| 2,2,4,6-Pentamethyl-heptane | 989 | 999 ^[4, 18, 38]^ | LRI, NIST |  |
| Decane | 1001 | 1000 ^[23,25]^ | LRI, NIST | 9, 37, 42, 59, |
| 2,2,4,4-Tetramethyl-octane | 1026 | 1021 ^[6, 12]^ | LRI, NIST |  |
| 2,6,7-Trimethyl-decane | 1051 | 1053 ^[16, 37, 60]^ | LRI, NIST | 37 |
| 2-Methyl-decane | 1064 | 1060 ^[25, 32]^ | LRI, NIST |  |
| 5-Undecene | 1077 | 1081 ^[39]^ | LRI, NIST |  |
| Undecane | 1100 | 1100 ^[21, 25]^ | LRI, NIST | 3, 9, 37, 66 |
| 2,8-dimethyl-4-methylene-nonane | 1126 |  | NIST |  |
| Pentyl-cyclohexane | 1134 | 1130 ^[25, 32]^ | LRI, NIST |  |
| 3-Methylene-undecane | 1184 | 1181 ^[4, 37]^ | LRI, NIST |  |
| Dodecane | 1200 | 1200 ^[23]^ | LRI, NIST | 3, 9, 37, 42 |
| 2,6,11-trimethyl dodecane | 1276 | 1275 ^[18, 50]^ | LRI, NIST | 9 |
| Tridecane | 1301 | 1300 ^[23]^ | LRI, NIST | 3, 37 |
| 2,3,5,8-tetramethyl-decane | 1322 | 1325 ^[4, 18]^ | LRI, NIST |  |
| Tetradecane | 1400 | 1400 ^[23]^ | LRI, NIST | 37, 46, 48 |
| Pentadecane | 1501 | 1500 ^[23]^ | LRI, NIST | 37 |
| 2,6,10-trimethyl- tetradecane | 1543 | 1543 ^[18]^ | LRI, NIST |  |
| Hexadecane | 1600 | 1600 ^[23]^ | LRI, NIST | 37 |
| Heptadecane | 1700 | 1700 ^[23]^ | LRI, NIST |  |
|  |  |  |  |  |
| Thiols |  |  |  |  |
| 2-Ethyl-1-hexanethiol | 1036 | 1032 ^[30]^ | LRI, NIST |  |
| 2-Methyl-2-heptanethiol | 1041 |  | NIST |  |
|  |  |  |  |  |
| Esters |  |  |  |  |
| Pentanoic acid,2,2,4-trimethyl-3-hydroxy-, isobutyl ester | 1346 |  | NIST |  |
| Carbamodithioic acid, diethyl-, methyl ester | 1373 | 1379 ^[63]^ | LRI, NIST |  |
| Dimethyl phtalate | 1444 | 1448 ^[8, 20]^ | LRI, NIST |  |
| Pentanoic acid, 2,2,4-trimethyl-3-carboxyisopropyl, isobutyl ester | 1585 |  | NIST |  |
|  |  |  |  |  |
| Lactone |  |  |  |  |
| γ-Nonalactone | 1358 | 1360 ^[10, 36, 56]^ | LRI, NIST | 11 |
|  |  |  |  |  |
| Acid |  |  |  |  |
| Dodecanoic acid | 1562 | 1568 ^[28, 36, 65]^ | LRI, NIST |  |
|  |  |  |  |  |
| Nitrile |  |  |  |  |
| 4-Cyano-cyclohexene | 1016 | 1012 ^[33, 47]^ | LRI, NIST |  |
|  |  |  |  |  |
| Azide |  |  |  |  |
| 2-Azido-2,4,4,6,6-pentamethylheptane | 1319 |  | NIST |  |
|  |  |  |  |  |
| Unknown |  |  |  |  |
| Unknown^d^ (RT=13.40 min) | 978 |  |  |  |
| Unknown^d^ (RT= 15.26 min) | 1023 |  |  |  |
| Unknown^d^ (RT= 16.51 min) | 1053 |  |  |  |
| Unknown^e^ (RT=17.96 min) | 1089 |  |  |  |
| Unknown^d^ (RT=18.15 min) | 1094 |  |  |  |
| Unknown^e^ (RT= 23.76 min) | 1244 |  |  |  |
| Unknown^e^ (RT=28.62 min) | 1387 |  |  |  |
| Unknown^e^ (RT=37.40 min) | 1681 |  |  |  |

^a^Linear Retention Indices calculated from the n-alkanes (C7-C30) run under the same GC-MS conditions as chicken muscle; ^b^Average LRI from the referenced literature on the TG-5SilMS column or equivalent; ^c^Method of Identification: National Institute of Standards and Technology database(NIST) and LRI; ^d^Compounds detected in Trial 1; ^e^Compounds detected in Trial 2.

References

1. Amanpour, A., Vandamme, J., Polat, S., Kelebek, H., Van Durme, J., & Selli, S. (2019). Non-thermal plasma effects on the lipoxygenase enzyme activity, aroma and phenolic profiles of olive oil. Innovative Food Science and Emerging Technologies, 54, 123-131. doi: 10.1016/j.ifset.2019.04.004
2. Ang, C. Y. W., Liu, F., & Sun, T. (1994). Development of a Dynamic Headspace GC Method for Assessing the Influence of Heating End-Point Temperature on Volatiles of Chicken Breast Meat. Journal of Agricultural and Food Chemistry, 42(11), 2493-2498. doi: 10.1021/jf00047a023
3. Byrne, D. V., Bredie, W. L. P., Mottram, D. S., & Martens, M. (2002). Sensory and chemical investigations on the effect of oven cooking on warmed-over flavour development in chicken meat. Meat Science, 61(2), 127-139. doi: 10.1016/S0309-1740(01)00171-1
4. Chen, F., Su, Y., Zhang, F., & Guo, Y. (2015). Low-temperature headspace-trap gas chromatography with mass spectrometry for the determination of trace volatile compounds from the fruit of Lycium barbarum L. Journal of Separation Science, 38(4), 670-676. doi: 10.1002/jssc.201400862
5. Cho, K. M., Lim, H. J., Kim, M. S., Kim, D. S., Hwang, C. E., Nam, S. H., . . . Shin, E. C. (2017). Time course effects of fermentation on fatty acid and volatile compound profiles of Cheonggukjang using new soybean cultivars. Journal of Food and Drug Analysis, 25(3), 637-653. doi: 10.1016/j.jfda.2016.07.006
6. Cordovez, V. D. C. (2016). Volatile-mediated interactions in the rhizosphere. Doctorate, Wageningen University, Netherlands Institute of Ecology.
7. Das, P. R., Kim, Y., Hong, S. J., & Eun, J. B. (2019). Profiling of volatile and non-phenolic metabolites—Amino acids, organic acids, and sugars of green tea extracts obtained by different extraction techniques. Food Chemistry, 296(January), 69-77. doi: 10.1016/j.foodchem.2019.05.194
8. Du, L., Li, J., Li, W., Li, Y., Li, T., & Xiao, D. (2014). Characterization of volatile compounds of pu-erh tea using solid-phase microextraction and simultaneous distillation-extraction coupled with gas chromatography-mass spectrometry. Food Research International, 57, 61-70. doi: 10.1016/j.foodres.2014.01.008
9. Duan, Y., Zheng, F., Chen, H., Huang, M., Xie, J., Chen, F., & Sun, B. (2015). Analysis of volatiles in Dezhou Braised Chicken by comprehensive two-dimensional gas chromatography/high resolution-time of flight mass spectrometry. LWT-Food Science and Technology, 60(2), 1235-1242. doi: 10.1016/j.lwt.2014.09.006
10. Fan, G., Sun, B., Fu, Z., Xia, Y., Huang, M., Xu, C., & Li, X. (2018). Analysis of Physicochemical Indices, Volatile Flavor Components, and Microbial Community of a Light-Flavor Daqu. Journal of the American Society of Brewing Chemists, 76(3), 209-218. doi: 10.1080/03610470.2018.1424402
11. Fan, M., Xiao, Q., Xie, J., Cheng, J., Sun, B., Du, W., . . . Wang, T. (2018). Aroma Compounds in Chicken Broths of Beijing Youji and Commercial Broilers. Journal of Agricultural and Food Chemistry, 66(39), 10242-10251. doi: 10.1021/acs.jafc.8b03297
12. Fernández, M., Hospital, X. F., Arias, K., & Hierro, E. (2016). Application of Pulsed Light to Sliced Cheese: Effect on Listeria Inactivation, Sensory Quality and Volatile Profile. Food and Bioprocess Technology, 9(8), 1335-1344. doi: 10.1007/s11947-016-1721-2
13. Gasser, U., & Grosch, W. (1990). Primary odorants of chicken broth - A comparative study with meat broths from cow and ox. Zeitschrift für Lebensmittel-Untersuchung und -Forschung, 190(1), 3-8. doi: 10.1007/BF01188254
14. Geeraerts, W., De Vuyst, L., Leroy, F., & Van Kerrebroeck, S. (2019). Monitoring of volatile production in cooked poultry products using selected ion flow tube-mass spectrometry. Food Research International, 119, 196-206. doi: 10.1016/j.foodres.2019.01.063
15. Gkarane, V., Brunton, N. P., Harrison, S. M., Gravador, R. S., Allen, P., Claffey, N. A., . . . Monahan, F. J. (2018). Volatile Profile of Grilled Lamb as Affected by Castration and Age at Slaughter in Two Breeds. Journal of Food Science, 83(10), 2466-2477. doi: https://doi.org/10.1111/1750-3841.14337
16. Gonzalez, M., Celis, A. M., Guevara-Suarez, M. I., Molina, J., & Carazzone, C. (2019). Yeast smell like what they eat: Analysis of Volatile Organic Compounds of Malassezia furfur in Growth Media Supplemented with Different Lipids. Molecules, 24(419), 1-19. doi: doi:10.3390/molecules24030419
17. Hong, S., Kang, W., Su, Y., & Guo, Y. (2013). Analysis of trace-level volatile compounds in fresh turf crop (Lolium perenne L.) by gas chromatography quadrupole time-of-flight mass spectrometry. Chinese Journal of Chemistry, 31(10), 1329-1335. doi: 10.1002/cjoc.201300414
18. Huang, X. H., Zheng, X., Chen, Z. H., Zhang, Y. Y., Du, M., Dong, X. P., . . . Zhu, B. W. (2019). Fresh and grilled eel volatile fingerprinting by e-Nose, GC-O, GC–MS and GC × GC-QTOF combined with purge and trap and solvent-assisted flavor evaporation. Food Research International, 115(July 2018), 32-43. doi: 10.1016/j.foodres.2018.07.056
19. Jerković, I., Kranjac, M., Marijanović, Z., Roje, M., & Jokić, S. (2019). Chemical Diversity of Headspace and Volatile Oil Composition of Two Brown Algae (Taonia atomaria and Padina pavonica) from the Adriatic Sea. Molecules. doi: 10.3390/molecules24030495
20. Jerković, I., Šuste, M., Željan, M., & Pilepić, K. H. (2012). Essential Oil Composition of Prasium majus from Croatia. Natural Product Communications, 7(7), 931-932.
21. Jin, Y., Deng, Y., Yue, J., Zhao, Y., Yu, W., Liu, Z., & Huang, H. (2015). Significant improvements in the characterization of volatile compound profiles in squid using simultaneous distillation-extraction and GC×GC-TOFMS. CYTA - Journal of Food, 13(3), 434-444. doi: 10.1080/19476337.2014.997798
22. Kerler, J., & Grosch, W. (1997). Character impact odorants of boiled chicken: Changes during refrigerated storage and reheating. Zeitschrift fuer Lebensmittel -Untersuchung und -Forschung, 205(3), 232-238. doi: 10.1007/s002170050157
23. Kondjoyan, N., & Berdagué, J.-L. (1996). A compilation of relative retention indices for the analysis of aromatic compounds. INRA:Theix, Station de Recherche sur la Viande, Sainte Genes Champanelle, France: Ed. du Laboratoire Flaveur.
24. Konoz, E., Abbasi, A., Parastar, H., Moazeni, R. S., & Jalali-Heravi, M. (2015). Analysis of olive fruit essential oil: Application of gas chromatography-mass spectrometry combined with chemometrics. International Journal of Food Properties, 18(2), 316-331. doi: 10.1080/10942912.2012.687797
25. Kotowska, U., Żalikowski, M., & Isidorov, V. A. (2012). HS-SPME/GC-MS analysis of volatile and semi-volatile organic compounds emitted from municipal sewage sludge. Environmental Monitoring and Assessment, 184(5), 2893-2907. doi: 10.1007/s10661-011-2158-8
26. Liu, J., Liu, M., He, C., Song, H., & Chen, F. (2015). Effect of thermal treatment on the flavor generation from Maillard reaction of xylose and chicken peptide. LWT - Food Science and Technology, 64(1), 316-325. doi: 10.1016/j.lwt.2015.05.061
27. Lu, S., Su, Y., & Guo, Y. (2011). Analysis of the volatile compounds in Senecio scandens Buch-Ham by gas chromatography-tandem mass spectrometry based on diversified scan technologies. European Journal of Mass Spectrometry, 17(4), 353-363. doi: 10.1255/ejms.1142
28. Madruga, M. S., Elmore, J. S., Oruna-Concha, M. J., Balagiannis, D., & Mottram, D. S. (2010). Determination of some water-soluble aroma precursors in goat meat and their enrolment on flavour profile of goat meat. Food Chemistry, 123(2), 513-520. doi: 10.1016/j.foodchem.2010.04.004
29. Menezes, A. M., Cruz Junior, C. A., Tanure, C. B., Peripolli, V., Castro, M. B., Louvandini, H., & McManus, C. (2016). Evaluation of carcass and muscle traits in Santa Ines female lambs finished with different agricultural products. Arquivo Brasileiro de Medicina Veterinária e Zootecnia, 68, 1259-1266.
30. Misra, B. B., Das, S. S., & Dey, S. (2013). Volatile profiling from heartwood of East Indian sandalwood tree. Journal of Pharmacy Research, 7(4), 299-303. doi: 10.1016/j.jopr.2013.04.030
31. Mitra, S., Burger, B. V., & Poddar-Sarkar, M. (2013). Headspace volatile oxylipins of eastern himalayan moss cyathophorella adiantum extracted by sample enrichment probe. Lipids, 48(10), 997-1004. doi: 10.1007/s11745-013-3807-1
32. Nadaf, M., Nasrabadi, M., & Halimi, M. (2012). GC-MS Analysis of n-Hexane Extract from Aerial Parts of Salvia nemorosa. 11(8), 1127-1130.
33. NIST. (2011). NIST/EPA/NIH Mass Spectral Database (Version 2.0 g, 2011)
34. Niu, Y., Wu, M., Xiao, Z., Chen, F., Zhu, J., & Zhu, G. (2016). Effect of Fatty Acids Profile with Thermal Oxidation of Chicken Fat on Characteristic Aroma of Chicken Flavors Assessed by Gas Chromatography-Mass Spectrometry and Descriptive Sensory Analysis. Food Science and Technology Research, 22(2), 245-254. doi: 10.3136/fstr.22.245
35. Nonaka, M., Black, D. R., & Pippen, E. L. (1967). Gas Chromatographic and Mass Spectral Analyses of Cooked Chicken Meat Volatiles. Journal of Agricultural and Food Chemistry, 15(4), 713-717. doi: 10.1021/jf60152a009
36. Pino, J. A., Mesa, J., Muñoz, Y., Martí, M. P., & Marbot, R. (2005). Volatile components from mango (Mangifera indica L.) cultivars. Journal of Agricultural and Food Chemistry, 53(6), 2213-2223. doi: 10.1021/jf0402633
37. Qi, J., Wang, H. h., Zhou, G. h., Xu, X. l., Li, X., Bai, Y., & Yu, X. b. (2018). Evaluation of the taste-active and volatile compounds in stewed meat from the Chinese yellow-feather chicken breed. International Journal of Food Properties, 20(3), S2579-S2595. doi: 10.1080/10942912.2017.1375514
38. Salvá, B. K., Fernández-Diez, A., Ramos, D. D., Caro, I., & Mateo, J. (2012). Chemical composition of alpaca (Vicugna pacos) charqui. Food Chemistry, 130(2), 329-334. doi: 10.1016/j.foodchem.2011.07.046
39. Saucier, C., Polidoro, A. d. S., dos Santos, A. L., Schneider, J. K., Caramão, E. B., & Jacques, R. A. (2014). Comprehensive two-dimensional gas chromatography with mass spectrometry applied to the analysis of volatiles in artichoke (Cynara scolymus L.) leaves. Industrial Crops and Products, 62, 507-514. doi: 10.1016/j.indcrop.2014.09.023
40. Schindler, S., Krings, U., Berger, R. G., & Orlien, V. (2010). Aroma development in high pressure treated beef and chicken meat compared to raw and heat treated. Meat Science, 86(2), 317-323. doi: 10.1016/j.meatsci.2010.04.036
41. Siegmund, B., & Pfannhauser, W. (1999). Changes of the volatile fraction of cooked chicken meat during chill storing. Results obtained by the electronic nose in comparison to GC-MS and GC olfactometry. Zeitschrift fuer Lebensmittel-Untersuchung und -Forschung A: Food Research and Technology, 208(5-6), 336-341. doi: 10.1007/s002170050426
42. Silva, F. A. P., Ferreira, V. C. S., Madruga, M. S., & Estévez, M. (2017). Aroma profile and consumer liking of salted and dried chicken meat: Effects of desalting and cooking methods. International Journal of Food Properties, 20(12), 2954-2965. doi: 10.1080/10942912.2016.1263653
43. Sohaib, M., Anjum, F. M., Arshad, M. S., Imran, M., Imran, A., & Hussain, S. (2017). Oxidative stability and lipid oxidation flavoring volatiles in antioxidants treated chicken meat patties during storage. Lipids in Health and Disease, 16(1), 1-10. doi: 10.1186/s12944-017-0426-5
44. Song, S., Yuan, L., Zhang, X., Hayat, K., Chen, H., Liu, F., . . . Niu, Y. (2013). Rapid measuring and modelling flavour quality changes of oxidised chicken fat by electronic nose profiles through the partial least squares regression analysis. Food Chemistry, 141(4), 4278-4288. doi: 10.1016/j.foodchem.2013.07.009
45. Takakura, Y., Mizushima, M., Hayashi, K., Masuzawa, T., & Nishimura, T. (2014). Characterization of the Key Aroma Compounds in Chicken Soup Stock Using Aroma Extract Dilution Analysis. Food Science and Technology Research, 20(1), 109-113. doi: 10.3136/fstr.20.109
46. Tang, J., Jin, Q. Z., Shen, G. H., Ho, C. T., & Chang, S. S. (1983). Isolation and Identification of Volatile Compounds from Fried Chicken. Journal of Agricultural and Food Chemistry, 31(6), 1287-1292. doi: 10.1021/jf00120a035
47. Tansawat, R. (2012). Chemical Characterization of Meat Related to Animal Diet. Doctor of Philosophy (Thesis), Utah State University, Logan, UT.
48. Taylor, D. L., & Larick, D. K. (1995a). Investigations into the Effect of Supercritical Carbon Dioxide Extraction on the Fatty Acid and Volatile Profiles of Cooked Chicken. Journal of Agricultural and Food Chemistry, 43(9), 2369-2374. doi: 10.1021/jf00057a010
49. Taylor, D. L., & Larick, D. K. (1995b). Volatile Content and Sensory Attributes of Supercritical Carbon Dioxide Extracts of Cooked Chicken Fat. Journal of Food Science, 60(6), 1197-1200. doi: 10.1111/j.1365-2621.1995.tb04554.x
50. Thang, T. D., Luu, H. V., Dung, V. C., Tuan, N. N., Hung, N. H., Dai, D. N., & Ogunwande, I. A. (2014). Chemical constituents of essential oils from the leaves and stem barks of four Vietnamese species of Fissistigma (Annonaceae). Natural Product Research, 28(3), 174-184. doi: 10.1080/14786419.2013.863199
51. Van Ba, H., Amna, T., & Hwang, I. (2013). Significant influence of particular unsaturated fatty acids and pH on the volatile compounds in meat-like model systems. Meat Science, 94(4), 480-488. doi: 10.1016/j.meatsci.2013.04.029
52. Van Ba, H., Oliveros, M. C., Ryu, K. S., & Hwang, L. (2010). Development of analysis condition and detection of volatile compounds from cooked Hanwoo beef by SPME-GC/MS analysis. Korean Journal for Food Science of Animal Resources, 30(1), 73-86.
53. Vasta, V., Luciano, G., Dimauro, C., Rohrle, F., Priolo, A., Monahan, F. J., & Moloney, A. P. (2011). The volatile profile of longissimus dorsi muscle of heifers fed pasture, pasture silage or cereal concentrate: Implication for dietary discrimination. Meat Science, 87(3), 282-289. doi: 10.1016/j.meatsci.2010.11.003
54. Verma, R. S., Padalia, R. C., Chauhan, A., Chanotiya, C. S., & Yadav, A. (2012). Chemical composition of the aliphatic compounds rich essential oil of Hypericum japonicum Thunb. ex Murray from India. Journal of Essential Oil Research, 24(6), 501-505. doi: 10.1080/10412905.2012.728082
55. Wang, C., Lv, S., Wu, Y., Lian, M., Gao, X., & Meng, Q. (2016). Study of aroma formation and transformation during the manufacturing process of Biluochun green tea in Yunnan Province by HS-SPME and GC-MS. Journal of the science of food and agriculture, 96(13), 4492-4498. doi: 10.1002/jsfa.7663
56. Wang, Y., Yang, C., Li, S., Yang, L., Wang, Y., Zhao, J., & Jiang, Q. (2009). Volatile characteristics of 50 peaches and nectarines evaluated by HP-SPME with GC-MS. Food Chemistry, 116(1), 356-364. doi: 10.1016/j.foodchem.2009.02.004
57. Watanabe, A., Ueda, Y., Higuchi, M., & Shiba, N. (2008). Analysis of volatile compounds in beef fat by dynamic-headspace solid-phase microextraction combined with gas chromatography-mass spectrometry. Journal of Food Science, 73(5), 420-425. doi: 10.1111/j.1750-3841.2008.00764.x
58. Wen, Y., Nie, J., Li, Z. G., Xu, X. Y., Wei, D., & Lee, M. R. (2014). The development of ultrasound-assisted extraction/dispersive liquid-liquid microextraction coupled with DSI-GC-IT/MS for analysis of essential oil from fresh flowers of Edgeworthia chrysantha Lindl. Analytical Methods, 6(10), 3345-3352. doi: 10.1039/c4ay00115j
59. Wettasinghe, M., Vasanthan, T., Temelli, F., & Swallow, K. (2001). Volatile flavour composition of cooked by-product blends of chicken, beef and pork: A quantitative GC-MS investigation. Food Research International, 34(2-3), 149-158. doi: 10.1016/S0963-9969(00)00146-0
60. www.pubchem.ncbi.nlm.nih.gov/. Retrieved 12/12/2019
61. Xiao, S., Zhang, W. G., Lee, E. J., Ma, C. W., & Ahn, D. U. (2011). Lipid and Protein Oxidation of Chicken Breast Rolls as Affected by Dietary Oxidation Levels and Packaging. Journal of Food Science, 76(4), 612-617. doi: 10.1111/j.1750-3841.2011.02137.x
62. Xu, X., Van Stee, L. L. P., Williams, J., Beens, J., Adahchour, M., Vreuls, R. J. J., . . . Lelieveld, J. (2003). Comprehensive two-dimensional gas chromatography (GC×GC) measurements of volatile organic compounds in the atmosphere. Atmospheric Chemistry and Physics, 3(3), 665-682. doi: 10.5194/acp-3-665-2003
63. Xu, Y., Liu, Y., Jiang, C., Zhang, C., Li, X., Zhu, D., & Li, J. (2014). Determination of volatile compounds in turbot (Psetta maxima) during refrigerated storage by headspace solid-phase microextraction and gas chromatography-mass spectrometry. Journal of the Science of Food and Agriculture, 94(12), 2464-2471. doi: 10.1002/jsfa.6581
64. Yan, J., Liu, X. B., Zhu, W. W., Zhong, X., Sun, Q., & Liang, Y. Z. (2014). Retention Indices for Identification of Aroma Compounds by GC: Development and Application of a Retention Index Database. Chromatographia, 78(1-2), 89-108. doi: 10.1007/s10337-014-2801-y
65. Yin, Z., Zhang, J., & Kang, W. (2016). Volatile components of Asparagus cochinchinensis stems. Chemistry of Natural Compounds, 52(6), 1116-1117. doi: 10.1007/s10600-016-1879-x
66. Zhang, M., Chen, X., Hayat, K., Duhoranimana, E., Zhang, X., Xia, S., . . . Xing, F. (2018). Characterization of odor-active compounds of chicken broth and improved flavor by thermal modulation in electrical stewpots. Food Research International, 109(January), 72-81. doi: 10.1016/j.foodres.2018.04.036
67. Zhuang, K., Wu, N., Wang, X., Wu, X., Wang, S., Long, X., & Wei, X. (2016). Effects of 3 Feeding Modes on the Volatile and Nonvolatile Compounds in the Edible Tissues of Female Chinese Mitten Crab (Eriocheir sinensis). Journal of Food Science, 81(4), S968-S981. doi: 10.1111/1750-3841.13229
